# Antithrombin concentrates may benefit cardiopulmonary bypass patients with suspected heparin resistance: A retrospective analysis of real-world data

**DOI:** 10.1016/j.heliyon.2023.e19497

**Published:** 2023-08-29

**Authors:** Stephen O. Bader, Xavier F. Marinaro, Glenda Stone, Kunal Lodaya, Jeffrey B. Spears, Aryeh Shander

**Affiliations:** a Heritage Valley Health System-Beaver, Department of Anesthesiology, USA; b Boston Strategic Partners, Inc., USA; c Grifols Shared Services North America, Inc., USA; d Englewood Health, Department of Anesthesiology, USA

**Keywords:** Antithrombin, Heparin resistance, Fresh frozen plasma, Cardiac surgery, Cardiopulmonary bypass, Mortality, Real-world data

## Abstract

**Background:**

Heparin resistance is a common complication of surgical patients requiring anticoagulation, such as those undergoing cardiopulmonary bypass (CPB). Treatments to address heparin resistance include supplementation of antithrombin (AT) or fresh frozen plasma (FFP). This retrospective database analysis compared key outcomes in suspected heparin-resistant patients undergoing CPB treated with AT or FFP.

**Methods:**

De-identified United States electronic health records (Cerner Health Facts®) were queried. International Classification of Diseases (ICD-9/10) codes were used to determine CPB procedures and FFP administration. AT administration was identified using medication data, while a combination of lab and medication data examining activated clotting times detected heparin resistance in FFP patients. Adult inpatients (≥18 years old) seen between 2001 and 2018 were included. Differences in mortality, intensive care unit (ICU) length of stay (LOS), and hospital-free days (using a 30-day post-discharge period) were assessed with univariate models as well as adjusted logistic regression models controlling for patient characteristics and Charlson Comorbidity Index (CCI) scores.

**Results:**

Of the 502 patients identified, 247 received AT and 255 received FFP. The FFP cohort was associated with a higher CCI compared to the AT cohort (3.3 ± 2.4 vs. 2.3 ± 2.0, *P* < .001). The AT cohort was associated with a 71% (Odds Ratio [OR]: 0.29, 95% Confidence Interval [CI]: *P* = .003) and 66% (OR: 0.34, 95% CI: *P* = .01) reduction in mortality when compared to FFP using univariate and adjusted logistic regression models, respectively. Similarly, use of AT also showed a 22% shorter ICU LOS (*P* = .02) and 10% more hospital-free days in the 30 days following discharge (*P* = .004) according to the univariate models, though statistical significance was absent within adjusted models in both ICU LOS (*P* = .08) and hospital-free days (*P* = .53).

**Conclusions:**

Compared to FFP, AT use suggests a reduction in the odds of mortality in suspected heparin-resistant patients undergoing CPB, though larger prospective studies are necessary to elucidate potential differences in hospital-free days or ICU LOS across treatment modalities.

## Highlights

• About 20% of cardiopulmonary bypass patients have an inadequate response to heparin
• Treatments include administration of antithrombin (AT) or fresh frozen plasma (FFP)
• Lower odds of mortality are suggested in AT over FFP supplementation before CPB
• Shorter lengths of hospital stay may be associated with AT supplementation

## 1 Introduction

Adequate anticoagulation is critical for the successful management of hemostatic and inflammatory responses that may result after cardiac procedures [1,2]. Heparin is an anticoagulant that enhances the binding and activity of antithrombin (AT), a small glycoprotein that inhibits the clotting process through enzyme inactivation [[3], [4], [5]]. Heparin resistance is a condition defined as the failure to achieve a targeted activated clotting time (ACT) of 400–480 s after a standard heparin dose, although a ‘standard’ heparin dose is ill-defined and ranges from less than 400 to 1200 U/kg of body weight. It is reported that approximately 22% of surgical patients requiring anticoagulation develop heparin resistance, such as those undergoing cardiac surgery involving cardiopulmonary bypass (CPB) [[6], [7], [8]]. Significantly decreased AT activity is more commonly associated with heparin resistance rather than a hereditary deficiency and increases the likelihood of clot formation as well as the risks of venous thrombosis and pulmonary embolism [3,9].

Factors contributing to decreased AT activity and heparin resistance include: (i) decreased plasma AT levels and greater hepatic AT clearance during cardiac procedures [3]; (ii) hereditary conditions, such as Type I and II hereditary AT III deficiency [7]; and (iii) secondary conditions affecting the degree to which the target ACT is achieved, such as liver failure, nephrotic syndrome, severe burns, trauma, and metastatic tumors [7,10]. Ranucci et al. reported a significant association between decreased AT activity in intensive care units (ICUs), and a higher incidence of allogeneic blood components use, blood loss, and renal dysfunction, as well as prolonged mechanical ventilation time, ICU stay, hospital mortality, and surgical re-exploration [7,10]. Additionally, the authors identified that decreased AT activity in ICU settings was also associated with low cardiac output syndrome, adverse neurologic events, and thromboembolic events [7,10].

In 2011, a recommendation from the Society of Thoracic Surgeons (STS) and the Society of Cardiovascular Anesthesiologists (SCA) stated with class 1A evidence that supplementation of AT concentrates immediately preceding a CPB procedure is indicated to reduce plasma transfusion in AT-mediated heparin-resistant (HR) patients [6]. Treatments for low AT levels and heparin resistance include the administration of either fresh frozen plasma (FFP), which contains AT among other blood components, or an AT concentrate to correct the deficiency [1]. AT concentrates are considered the safer alternative due to their association with low incidences of adverse events and allergic reactions, the fiftyfold decrease in volume AT concentrates require to transfuse an equal dose of AT as FFP [11], and the higher likelihood FFP has for potential infectious disease transmission [1]. However, the comparative effect of AT concentrates versus FFP on morbidities and hospital mortality is largely unknown. This retrospective study aims to compare key patient outcomes in patients with suspected HR undergoing cardiac surgeries requiring CPB treated with FFP versus AT concentrates.

## 2 Materials and methods

### 2.1 Study design/cohort selection

This retrospective cohort analysis was conducted using a de-identified, U.S. electronic health record (EHR) database (Cerner Health Facts®; Cerner, Kansas City, MO) which contains patient encounter data from >700 hospitals nationwide. Data were analyzed from heparin-resistant patients aged ≥18 years who underwent inpatient cardiac surgery requiring CPB between 2001 and 2018 and received either AT concentrates or FFP during their stay. Records showing that patients received both FFP and AT concentrates or had missing/unknown sex were excluded from the study. This study was performed under an exempt status granted by the Institutional Review Board (IRB) defined in 45 Code of Federal Regulations (CFR) 46.102 and adheres to applicable STROBE guidelines.

#### 2.1.1 FFP cohort

International classification of diseases (ICD-9/10) codes (Supplemental Table 1) were used to identify CPB procedures, and heparin resistance for FFP patients was determined from lab and medication data based on previously established methods [[3], [4], [5], [6]] described in detail below.

**Fig. 1:**
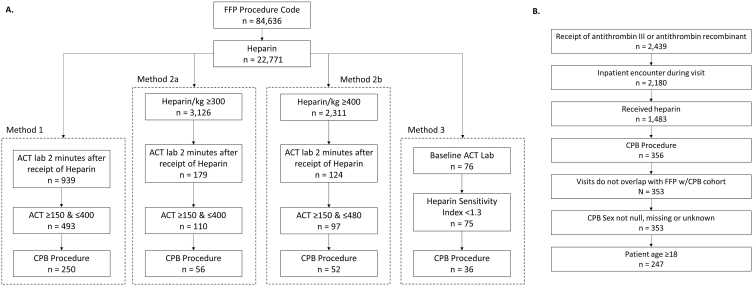
***Schematic showing attrition methods for FFP and AT cohorts***. A) Schematic showing different methods used to obtain the final FFP cohort number. CPB patients receiving FFP were distinguished by using International Disease Classification (ICD-9/10) procedure codes, followed by heparin use during surgery. Patients who fulfilled criteria in either Method 1, Method 2a, Method 2b, or Method 3 comprised the final FFP cohort. B) Schematic showing the method to obtain the final AT cohort number. CPB patients receiving AT were distinguished first by whether the AT was purified or recombinant, then whether patients had an inpatient encounter during visits followed by heparin administration during a CPB procedure. Patients with unknown or missing sex information, under 18, and whose visits overlapped with any patients in the FFP cohort were excluded. ACT, activated clotting time; AT, antithrombin; CPB, cardiopulmonary bypass; FFP, fresh frozen plasma.

Several literature-supported methods [5,8,12,13] were utilized to identify CPB patients with suspected HR receiving FFP (Fig. 1A; Supplemental Table 2). Method 1 consisted of identifying patient data containing an FFP procedure code (Supplemental Table 1), a CPB procedure code (Supplemental Table 1), patients who received heparin, an ACT lab test ≥2 min after the receipt of heparin, and an ACT between 150 and 400 s (Fig. 1A; Supplemental Table 2). Method 2a encompassed identifying patient data containing an FFP and CPB procedure code, patients who received heparin, an ACT lab test ≥2 min after the receipt of heparin, a heparin dose per kg of ≥300, and an ACT between 150 and 400 s (Fig. 1A; Supplemental Table 2). Method 2b involved identifying patient data with an FFP procedure code, a CPB procedure code, patients who received heparin, an ACT lab test ≥2 min after the receipt of heparin, a heparin dose per kg ≥ 400, and an ACT between 150 and 480 s. Finally, Method 3 included patient data with an FFP and CPB procedure code, patients who received heparin, a baseline ACT lab, and a heparin sensitivity index, calculated by the difference between baseline ACT and ACT after heparin administration divided by the heparin loading dose per kilogram [7], of <1.3 (Fig. 1A; Supplemental Table 2).

#### 2.1.2 AT concentrate cohort

The AT concentrate cohort comprised patients who: (i) received AT concentrates (determined by brand and generic names), (ii) had an inpatient encounter during a hospital visit, (iii) had a CPB procedure code (Supplemental Table 1), and (iv) received heparin (Fig. 1B; Supplemental Table 2).

### 2.2 Statistical analysis

We explored patient characteristics including age, sex, ethnicity, Charlson Comorbidity Index (CCI) scores, acute physiology scores (APS), as well as hospital characteristics such as bed size and teaching status. CCI is the most widely used comorbidity index, and utilizes a weighted sum that predicts the risk of death within one year of hospitalization for patients with any combination of a certain 19 specific comorbid conditions [14]. In-hospital mortality was analyzed as the primary outcome. Secondary outcomes included hospital-free days and ICU length-of-stay (LOS). Hospital-free days were defined as the number of days that a patient was not hospitalized following discharge for up to 30 days and was a continuous outcome. ICU LOS was defined from the day and time of the first lab test/medication order documenting an ICU care setting to a discharge entry noting an ICU care setting; or as the last day/time of a lab/medication order/microbiology test/encounter with a documented ICU care setting as a continuous outcome. Patients who died at any point during their hospital stay were excluded from ICU LOS classification.

Differences between AT concentrate and FFP cohorts’ patient characteristics were analyzed via chi-squared tests for categorical variables and t-tests for continuous variables. Outcomes of interest were analyzed in both univariate and multivariable analyses. The multivariable analysis was adjusted for differences in patient characteristics, including age, sex, ethnicity, index year (between 2001 and 2018), and CCI score. Logistic regression was utilized to examine the association between exposures and binary outcomes, while a Generalized Linear Model (GLM) with log link was utilized to model the association between each exposure and continuous outcomes. Hospital mortality was categorized as a binary outcome to estimate the effect of each exposure of interest on mortality at the index visit. Secondary (exploratory) exposures included ventilation duration, red blood cell (RBC) transfusion, and renal replacement therapy (RRT). All statistical analyses were performed based on an alpha level of 0.05. SAS version 9.4 was used for these analyses (SAS Institute Inc., Cary, NC).

## 3 Results

**Fig. 2:**
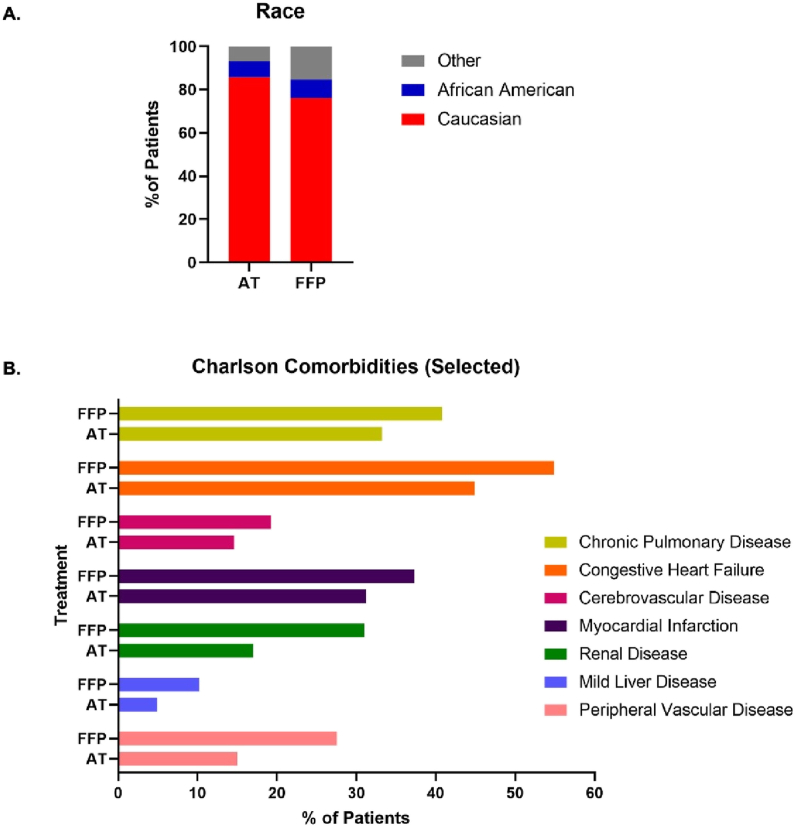
**(In color): *Patients of the FFP and AT cohorts shown by race and selected Charlson Comorbidities***. A) Patients in the AT or FFP cohorts shown by ethnicity (African American, Caucasian, and Other*). B) Patients in the AT or FFP cohorts shown by selected Charlson comorbidities. Data show the percentage of patients identified per patient characteristic as a function of treatment type. *Asian/Pacific Islander, Hispanic, Other, and Not Specified. AT, antithrombin; FFP, fresh frozen plasma.

Out of the 502 patients who met the inclusion criteria, 247 (49.2%) received AT concentrates and 255 (50.8%) received FFP (Fig. 1A and B; Supplemental Table 2). Both cohorts were well-balanced by age (AT: 64.4 ± 12.4; FFP: 63.6 ± 13.5) and sex (AT: 30.4% female; FFP: 31.8% female), but significant differences were seen in ethnicity (*P* = .008; AT: 85.8% Caucasian; FFP: 76.1% Caucasian) and payer type (*P* < .001) (Table 1; Fig. 2A). Additionally, the FFP cohort demonstrated a significantly higher CCI (Median [25th, 75th] FFP: 3 [2,5]; AT: 2 [1,3]; *P* < .001) (Fig. 2B; Supplemental Table 3) and APS scores (Median [25th, 75th] FFP: 10 [4,15]; AT: 5 [0,12]; *P* < .001) compared to the AT concentrate cohort (Table 1; Fig. 2B; Supplemental Table 3). Compared to AT concentrate patients, the FFP cohort had a higher proportion of patients on ventilation support overall (FFP: 30.6%, AT: 7.3%) and ventilation support >96 h (FFP: 15.3%, AT: 2.4%) (Table 2). Similarly, compared to AT concentrate patients, the FFP cohort had a higher proportion of RRT (FFP: 11.4%, AT: 4.5%) and RBC transfusions (FFP: 83.1%, AT: 42.1%) (Table 2).

**Table 1:** Patient characteristics.

Characteristic	AT (N = 247)	FFP (N = 255)	*P-*value^*a*^
Age, year			
Mean ± SD	64.4 ± 12.4	63.6 ± 13.5	.46
Median [25th, 75th]	66 [58, 73]	66 [54, 75]	
Min to Max	18 to 87	19 to 86	
**Age group**			
18–49	32 (13.0)	37 (14.5)	.84
50–64	78 (31.6)	82 (32.2)	
65+	137 (55.5)	136 (53.3)	
**Sex**			
Male	172 (69.6)	174 (68.2)	.74
Female	75 (30.4)	81 (31.8)	
**Ethnicity**			
Caucasian	212 (85.8)	194 (76.1)	.008
African American	18 (7.3)	22 (8.6)	
Other^a^	17 (6.9)	39 (15.3)	
**Payer**			
Null or Unknown	200 (81.0)	28 (11.0)	<.001
Medicare	27 (10.9)	126 (49.4)	
Commercial	12 (4.9)	63 (24.7)	
Medicaid	1 (0.4)	21 (8.2)	
Self	1 (0.4)	10 (3.9)	
Other, Government, or Military	3 (1.2)	6 (2.4)	
Other, NGO or Worker's Compensation	3 (1.2)	1 (0.4)	
**Admission source**			
Physician or Clinical Referral	94 (38.1)	129 (50.6)	<.001
Hospital or Facility Transfer	81 (32.8)	41 (16.1)	
Emergency Room	57 (23.1)	52 (20.4)	
Not Specified	15 (6.1)	33 (12.9)	
**Admission type**			
Emergency/Urgent	150 (60.7)	139 (54.5)	<.001
Elective	80 (32.4)	115 (45.1)	
Not Specified	17 (6.9)	1 (0.4)	
**Index Year**			
2001–2006	130 (52.6)	5 (2.0)	<.001
2007–2012	64 (25.9)	85 (33.3)	
2012–2017	53 (21.5)	165 (64.7)	
**Charlson Comorbidity Index**			
Mean ± SD	2.3 ± 2.0	3.3 ± 2.4	<.001
Median [25th, 75th]	2 [1,3]	3 [2,5]	
Min to Max	0 to 10	0 to 12	
**APS score**			
Mean ± SD	6.9 ± 7.5	13.0 ± 10.9	<.001
Median [25th, 75th]	5 [0, 12]	10 [4,15]	
Min to Max	0 to 29	0 to 47	

Data are numbers (percentages) unless otherwise indicated. ^*a*^T-tests were conducted for continuous data while chi-square tests were conducted for categorical data.

a Other categories consisted of Asian/Pacific Islander, Hispanic, Other, and Not Specified ethnicities. APS, acute physiology score; AT, antithrombin; FFP, fresh frozen plasma; NGO, non-governmental organization; SD, standard deviation.

**Table 2:** Outcomes and exposures.

Characteristic	AT (N = 247)	FFP (N = 255)
Primary outcome		
Mortality		
Yes	8 (3.2)	26 (10.2)
No	239 (96.8)	229 (89.8)
**Secondary outcomes**		
Hospital LOS, days^a^		
N	239	229
Mean ± SD	15.2 ± 14.5	15.6 ± 11.5
Median [25th, 75th]	11.5 [7.5, 16.9]	12.8 [8.2, 18.1]
Min to Max	3.3 to 129.1	3.4 to 78.7
ICU LOS, days^b^		
N	170	108
Mean ± SD	9.0 ± 11.5	11.6 ± 11.8
Median [25th, 75th]	6.6 [3.2, 10.5]	8.7 [4.3, 13.7]
Min to Max	0.8 to 115.7	0.0 to 75.9
Hospital free days in the first 30 days from surgery		
Mean ± SD	19.6 ± 7.8	16.2 ± 8.7
Median [25th, 75th]	23 [18,25]	20 [11,23]
Min to Max	0 to 27	0 to 27
**Secondary exposures**		
Ventilation duration		
≤96 h.	11 (4.5)	35 (13.7)
>96 h.	6 (2.4)	39 (15.3)
UNKNOWN^c^	1 (0.4)	4 (1.6)
NONE	229 (92.7)	177 (69.4)
RBC transfusion		
Yes	104 (42.1)	212 (83.1)
No	143 (57.9)	43 (16.9)
RRT		
Yes	11 (4.5)	29 (11.4)
No	236 (95.5)	226 (88.6)

Data are numbers (percentages) unless otherwise indicated.

a LOS is shown for visits without index visit mortality.

b ICU LOS is shown for visits without mortality and an ICU stay.

c Unknown visits had ventilation, but time was not indicated in the ICD code. AT, antithrombin; FFP, fresh frozen plasma; International Disease Classification; ICU, intensive care unit; LOS, length of stay; RBC, red blood cell; RRT, renal replacement therapy; ICD, SD, standard deviation.

**Fig. 3:**
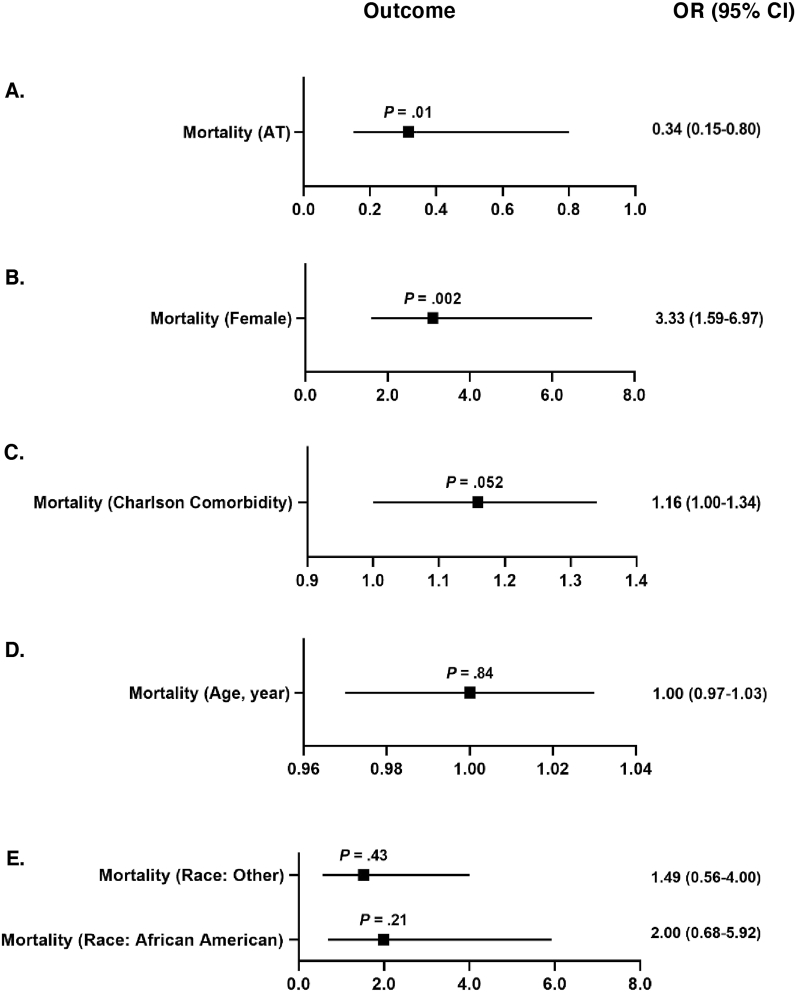
***Adjusted mortality outcomes using logistic regression***. Adjusted mortality outcomes using logistic regression. A) Likelihood of mortality in AT patients versus FFP patients (*reference*). B) Likelihood of mortality of female patients versus male patients (*reference*). C) Likelihood of mortality when adjusting for Charlson comorbidities. D) Likelihood of mortality when stratified by age. E) Likelihood or mortality in patients defined as Other* or African American versus Caucasian patients (*reference*). *Asian/Pacific Islander, Hispanic, Other, and Not Specified. OR, odds ratio; CI, confidence interval; AT, antithrombin; FFP, fresh frozen plasma.

The risk-adjusted model showed that the AT concentrate cohort had a 71% reduction in the odds of mortality compared to the FFP cohort (Odds Ratio [OR]: 0.29; 95% Confidence Interval [CI]: 0.13–0.66, *P* = .003) (Fig. 3A; Table 3; Supplemental Table 4). A similar result was observed within the unadjusted univariate analysis (OR: 0.34; 95% CI: 0.15–0.80, *P* = .01) (Fig. 3B–E; Table 3; Supplemental Table 4). The risk-adjusted model did not report statistically significant findings for hospital-free days (*P* = .08) (Table 4; Supplemental Table 5), but the univariate analysis found 10% more hospital-free days in the first 30 days from surgery in the AT concentrate cohort compared to the FFP cohort (OR: 1.10; 95% CI: 1.03–1.18, *P* = .004) (Table 4; Supplemental Table 5). Similarly, no significance was found after adjusting for these same exposures within the generalized linear model (GLM) for ICU LOS (*P* = .53) (Table 5; Supplemental Table 6), but the univariate analysis suggested the AT concentrate cohort had a significantly lower ICU LOS when compared to the FFP cohort (OR: 0.78; 95% CI: 0.63–0.96, *P* = .02).

**Table 3:** Mortality model (N = 502).

Characteristic	Model 1: Univariate Analyses		Model 2: Patient Characteristics and Charlson^a^	
	OR (95% CI)	*P-*value	OR (95% CI)	*P-*value
AT or FFP				
AT	0.29 (0.13–0.66)	.003	0.34 (0.15–0.80)	.01
FFP	*Reference*		*Reference*	
Age, year	1.00 (0.97–1.03)	.93	1.00 (0.97–1.03)	.84
Sex				
Male	*Reference*		*Reference*	
Female	3.49 (1.71–7.10)	*<* .001	3.33 (1.59–6.97)	.002
Ethnicity				
Caucasian	*Reference*		*Reference*	
African American	2.38 (0.85–6.64)	.10	2.00 (0.68–5.92)	.21
Other	2.00 (0.78–5.14)	.15	1.49 (0.56–4.00)	.43
Charlson Comorbidity Index	1.21 (1.06–1.39)	.004	1.16 (1.00–1.34)	.05

a Logistic regression with mortality as a binary outcome was used in Model 2. AT, antithrombin; CCI, Charlson Cormorbidity Index; CI, confidence interval; FFP, fresh frozen plasma; GLM, generalized linear model; OR, odds ratio.

**Table 4:** Hospital free days model (N = 502).

Characteristic	Model 1: Univariate Analyses		Model 2: Patient Characteristics and Charlson^a^	
	Hospital Free Days Ratio (95% CI)	*P-*value	Hospital Free Days Ratio (95% CI)	*P-*value
AT or FFP				
AT	1.10 (1.03–1.18)	.004	1.06 (0.99–1.13)	.08
FFP	*Reference*	*Reference*		
**Age, year**	1.00 (1.00–1.00)	.86	1.00 (1.00–1.00)	.56
**Sex**				
Male	*Reference*	*Reference*		
Female	0.97 (0.90–1.05)	.43	0.97 (0.91–1.05)	.49
**Ethnicity**				
Caucasian	*Reference*	*Reference*		
African American	0.86 (0.76–0.98)	.03	0.89 (0.78–1.01)	.06
Other	0.99 (0.89–1.10)	.81	1.02 (0.92–1.13)	.72
**CCI**	0.95 (0.94–0.97)	<.001	0.95 (0.94–0.97)	<.001

a GLMs with gamma distribution and log link function was used in Model 2. AT, antithrombin; CCI, Charlson Comorbidity Index; CI, confidence interval; FFP, fresh frozen plasma; GLM, generalized linear model.

**Table 5:** Intensive care unit length of stay model (N = 278).

Characteristic	Model 1: Univariate Analyses		Model 2: Patient Characteristics and Charlson^a^	
	ICU LOS Ratio (95% CI)	*P-*value	ICU LOS Ratio (95% CI)	*P-*value
AT or FFP				
AT	0.78 (0.63–0.96)	.02	0.93 (0.73–1.17)	.53
FFP	*Reference*		*Reference*	
Age, year	1.01 (1.00–1.01)	.16	1.00 (1.00–1.01)	.35
Sex				
Male	*Reference*		*Reference*	
Female	1.30 (1.04–1.63)	.02	1.21 (0.97–1.51)	.09
Ethnicity				
Caucasian	*Reference*		*Reference*	
African American	0.86 (0.55–1.34)	.51	0.92 (0.59–1.41)	.69
Other	1.07 (0.77–1.49)	.67	1.15 (0.84–1.58)	.39
Charlson Comorbidity Index	1.13 (1.08–1.18)	<.001	1.12 (1.06–1.17)	<.001

a GLMs with gamma distribution and log link function was used in Model 2. AT, antithrombin; CCI, Charlson Comorbidity Index; CI, confidence interval; FFP, fresh frozen plasma; GLM, generalized linear model; ICU, intensive care unit; LOS, length of stay.

## 4 Discussion

In this retrospective database study, we compared the effect of AT concentrates versus FFP on in-hospital mortality, hospital-free days, and ICU LOS, among suspected HR patients undergoing cardiac surgery requiring CPB. These real-world data suggest that the use of AT concentrate treatment in this patient population may lower the odds of in-hospital mortality when compared to those using FFP treatment. There are possible associations of more hospital-free days and fewer ICU days in this patient population with the supplementation of AT concentrate over FFP, however, future multivariate analyses are required to confirm these correlations. Importantly, HR patients undergoing CPB procedures often present with comorbidities such as peripheral vascular disease, cerebrovascular disease, and rheumatic disease that may play a role in perpetuating adverse treatment and disease outcomes.

Differences in patient demographics among the cohorts were found among a few baseline characteristics. It appears that a significantly larger portion of the FFP cohort was funded by Medicare/Medicaid, however, the AT concentrate cohort shows a drastically larger percentage of its cohort to have a “Null/Unknown” payer type. It may be possible that there were more Medicare/Medicaid patients in the AT cohort that exist within this “Null/Unknown” population. Although there is a distinct difference in geographic spread between cohorts, it should be noted that the Cerner Health Facts database groups patients into two very broad geographic regions (Northeast/South and Midwest/West of the U.S.) and that this generalizability of regional data makes it difficult to draw many conclusions based on geography. It was observed that FFP was used significantly more within 2012–2017 versus 2001–2006. Increased consumption at the tail end of our study period could potentially be due to the underutilization of patient blood management modalities to treat bleeding disorders and coagulopathies, along with the lack of strict institutional guidelines that trigger blood product transfusion [16]. Comorbidities were generally more prevalent among the FFP cohort, initially placing this cohort at a higher risk. Our risk-adjusted model takes these differences into account, yet the results of the univariate analyses require further research to confirm their conclusions.

Differences among secondary exposures were observed between the two cohorts. The FFP cohort showed a higher prevalence of RBC transfusions and ventilation support during their visits. The adjusted model considers CCI scores, which accounts for certain comorbidities that may contribute to the need for these transfusions. The STS Clinical Practice Guidelines state that patients who have better preserved postoperative hemostatic profiles after the physiological stresses of CPB tend to have less bleeding and blood transfusions. It is possible that these FFP patients were more likely to have poorer postoperative hemostatic profiles that may have resulted in the need for an RBC transfusion.

Currently, there are four main treatments aimed at achieving adequate anticoagulation in cardiac procedures such as CPB or extracorporeal membrane oxygenation (ECMO), when standard-of-care heparin fails to achieve the target ACT [2]. These include treatment with additional heparin, supplementation with an AT concentrate (recombinant or purified) or FFP, treatment with a different anticoagulant, or no additional treatment [7]. Guidelines from the Society of Thoracic Surgeons and the Society of Cardiovascular Anesthesiologists recommend the use of AT concentrates in cardiac surgeries where patients exhibit an AT-dependent heparin resistance mechanism [6]. Alternatively, guidelines indicate the use of FFP in cardiac surgeries if decreased AT levels, along with low coagulation factor levels, bleeding complications, or low fibrinogen levels, are identified [6]. In one study, 82% of examined centers used heparin combined with AT concentrates, FFP, or pooled supplementation during ECMO procedures, suggesting that supplementation is commonplace in clinical practice [2,17]. Another study and review found that clinicians treated cardiac patients with additional heparin doses as high as 1200 U/kg, relative to typical doses (300–400 U/kg plus additional doses) to achieve or maintain the ACT [7,18]. Even when using high doses of heparin, ACT levels during surgery may fail to reach target levels [7].

Several studies have shown that AT concentrates are effective at improving heparin responsiveness, as measured by the ACT in various cardiac surgeries including unstable angina and CPB, using a 50-fold lower volume than that required with FFP use [1,[15], [18], [19], [20]]. Since only a limited number of studies have demonstrated the effectiveness of AT concentrates to reduce hemostatic activation, bleeding complications, and other clinical outcome improvements beyond the target ACT, this area warrants further research [7,20,21]. A prospective double-blind placebo-controlled 20 patient study found no clinical benefit of AT supplementation in cardiac procedures relative to a saline placebo in preventing blood loss, fibrinolytic activity, or thrombin generation; however, heparin resistance was not a required inclusion parameter for participating patients [22]. A 2018 study estimated that the average cost of AT supplementation per patient was $3665 USD [2]. Another study that same year noted that a single vial of 500-U or 1000-U AT concentrate ─ doses commonly administered during cardiac surgery ─ cost $2330 or $4660 USD, respectively [23]. The results of our study suggest that patients with suspected HR undergoing CPB may derive some clinical benefit from AT supplementation, however, future studies understanding the economic impact from a variety of perspectives (e.g., patient, hospital, payer, society) are warranted.

Even more limited than studies assessing the efficacy of AT concentrates are those addressing FFP in CPB or similar procedures [1,7,22]. One study in 2018 found that FFP increased the ACT to the therapeutic target in HR patients undergoing ECMO, but patients had significantly higher bleeding complications and decreased survival relative to non-HR patients [22]. FFP use has been associated with transfusion-related and procedural risks from significant time delays due to the thawing period [1,7]. An alternative option, but less commonly employed by clinicians, is to continue CPB procedures without attaining the ACT target, using heparin doses based on a patient's weight [7].

Large database analyses, including this one, have limitations due to available data. In this study, the database only reported the date for CPB and FFP making it impossible to determine the exact timing of clinical events or surgical duration. Additionally, information on the number of FFP units and AT concentrate dose was not available. This demonstrates problems related to the poor, real-time interoperability between EHR and blood bank data that have been previously recognized [24]. The demographics and geography of the two cohorts were slightly imbalanced at baseline, with the FFP cohort at a generally higher overall risk. While the adjusted model accounts for this, it is still important to acknowledge the limitation that these differences may have affected unadjusted outcome at the individual and/or community levels. ACT and AT labs were not well documented in the AT concentrate cohort making lab comparisons difficult. The database did not contain physician notes so the exact reason for treating heparin resistance with FFP or AT concentrates is unknown and it is possible that sub-clinical HR patients were not identified. While our methods identified suspected HR patients in the AT concentrate and FFP cohorts, clinical rationale for AT concentrate and FFP treatment is uncertain. Although heparin resistance is the most logical explanation for the supplementation of AT concentrates or FFP based on our inclusion criteria, it is however possible that FFP or AT concentrate could have also been administered to correct other blood disorders. Further, although it is agreed that failure to achieve a targeted ACT after sufficient heparin administration qualifies for the labeling of heparin resistance, prior literature and retrospective studies on HR patients have stated that an official, definitive consensus for heparin resistance is currently lacking, and diagnosis, dosage, treatment, and appropriate target levels are all ill-defined [1]. Previous literature has inconsistently claimed a wide range from less than 400 to 1200 U/kg of body weight as the accepted “standard” dose of heparin. A recently published review article proposes a more structured definition, arguing that heparin resistance for patients undergoing CPB is often characterized by the need for a heparin dose of ≥500 U/kg to achieve an ACT of 400–480 s [25]. Unfortunately, our analysis was complete at the time this article was published. Nonetheless, a widely accepted definition of this condition is warranted.

While previous studies have assessed the efficacy and safety of AT concentrates and FFP individually, to date, there are no clinical trials comparing AT concentrates versus FFP in CPB or similar cardiac procedures, much less in HR cardiac patients. This study suggests potential benefits of AT concentrates versus FFP in patients with suspected HR undergoing CPB, particularly in the odds of mortality. Potential for decreased hospital resource utilization, despite its high initial costs, may be possible with the supplementation of AT concentrates over FFP, but future studies are warranted to confirm this. Future randomized controlled trials utilizing larger sample sizes comparing AT concentrates versus FFP versus a saline placebo, specifically in HR patients, would be beneficial to further examine the effects of both treatments on this targeted population in a controlled setting.

## Author contribution statement

Stephen O. Bader; Kunal Lodaya: Conceived and designed the experiments; Analyzed and interpreted the data; Contributed reagents, materials, analysis tools or data; Wrote the paper.

Xavier F. Marinaro: Conceived and designed the experiments; Performed the experiments; Analyzed and interpreted the data; Contributed reagents, materials, analysis tools or data.

Glenda Stone; Jeffrey B. Spears; Aryeh Shander; Conceived and designed the experiments; Analyzed and interpreted the data; Contributed reagents, materials, analysis tools or data.

## Data availability statement

Data will be made available on request.

## Funding

Financial support for this study was provided by 10.13039/501100016387Grifols Shared Services North America, Inc.

## Declaration of competing interest

The authors declare the following financial interests/personal relationships which may be considered as potential competing interests.

Stephen Bader reports financial support was provided by Grifols Shared Services North America, Inc. Aryeh Shander reports financial support was provided by Grifols Shared Services North America, Inc. Xavier Marinaro reports statistical analysis was provided by Boston Strategic Partners Inc. Kunal Lodaya reports statistical analysis was provided by Boston Strategic Partners Inc. Consultancy with Grifols Shared Services North America, Inc. -SB and AS; Employee of Boston Strategic Partners, Inc. when this study was conducted. -XM Employee of Grifols Shared Services North America, Inc. -GS and JS.
