# Supplementary material for: Antithrombin concentrates may benefit cardiopulmonary bypass patients with suspected heparin resistance: A retrospective analysis of real-world data
Source: Heliyon. 2023 Aug 29;9(9):e19497. doi: 10.1016/j.heliyon.2023.e19497 (PMC10558716; doi:10.1016/j.heliyon.2023.e19497)
Supplement: Multimedia component 1 [file mmc1.docx]

STROBE Statement—checklist of items that should be included in reports of cohort observational studies

|  | **Item No** | **Recommendation** | **Check** |
| --- | --- | --- | --- |
| **Title and Abstract** | 1 | (*a*) Indicate the study’s design with a commonly used term in the title or the abstract | *The title describes the study design as “A retrospective analysis”* |
|  |  | (*b*) Provide in the abstract an informative and balanced summary of what was done and what was found | *The abstract describes the methods and findings* |
| **Introduction** | | |  |
| Background/Rationale | 2 | Explain the scientific background and rationale for the investigation being reported | *The background and rationale are described throughout the Introduction* |
| Objectives | 3 | State specific objectives, including any prespecified hypotheses | *The aims and prespecified hypotheses based on published literature are stated in the last paragraph of the Introduction* |
| **Methods** | | |  |
| Study Design | 4 | Present key elements of study design early in the paper | *The study design is stated in the ‘Study Design/Cohort Selection’ subsection of the Methods* |
| Setting | 5 | Describe the setting, locations, and relevant dates, including periods of recruitment, exposure, follow-up, and data collection | *The setting and timing of the study are stated in the Methods* |
| Participants | 6 | (*a*) *Cohort study*—Give the eligibility criteria, and the sources and methods of selection of participants. Describe methods of follow-up | *Eligibility criteria is outlined in the ‘Study Design/Cohort Selection’ subsection of the Methods. Patient selection was described in the ‘FFP Cohort’ and ‘AT Cohort’ paragraphs of the Methods* |
|  |  | (*b*) *Cohort study*—For matched studies, give matching criteria and number of exposed and unexposed | *Not applicable* |
| Variables | 7 | Clearly define all outcomes, exposures, predictors, potential confounders, and effect modifiers. Give diagnostic criteria, if applicable | *Measured outcomes are outlined in the ‘Statistical Analysis’ subsection of the Methods. Potential confounders and modifiers are studied* |
| Data Sources/ Measurement | 8 | For each variable of interest, give sources of data and details of methods of assessment (measurement). Describe comparability of assessment methods if there is more than one group | *Data was sourced from an Electronic Health Record database as stated in the ‘Study Design/Cohort Selection’ subsection of the Methods. The details of the assessment methods varied between cohorts, and were outlined in their respective cohort subsections in the Methods* |
| Bias | 9 | Describe any efforts to address potential sources of bias | *Bias was addressed in the Methods through de-identified data and the exclusion of patients with missing demographic information* |
| Study Size | 10 | Explain how the study size was arrived at | *Sample determination was described in the ‘Study Design/Cohort Selection’ subsection of the Methods. Records of all patients satisfying the inclusion criteria were analyzed.* |
| Quantitative Variables | 11 | Explain how quantitative variables were handled in the analyses. If applicable, describe which groupings were chosen and why | *Quantitative variables were explained in the ‘Statistical Analysis’ subsection of the Methods* |
| Statistical Methods | 12 | (*a*) Describe all statistical methods, including those used to control for confounding | *Statistical methods were outlined in the ‘Statistical Analysis’ subsection of the Methods* |
|  |  | (*b*) Describe any methods used to examine subgroups and interactions | *Statistical methods were outlined in the ‘Statistical Analysis’ subsection of the Methods* |
|  |  | (*c*) Explain how missing data were addressed | *Cases with missing data were excluded* |
|  |  | (*d*) *Cohort study*—If applicable, explain how loss to follow-up was addressed | *Not applicable* |
|  |  | (*e*) Describe any sensitivity analyses | *Not applicable* |
| **Results** | | |  |
| Participants | 13 | (a) Report numbers of individuals at each stage of study—e.g., numbers potentially eligible, examined for eligibility, confirmed eligible, included in the study, completing follow-up, and analyzed | *The number of patients included in each respective cohort was explained in paragraph 1 of the Results* |
|  |  | (b) Give reasons for non-participation at each stage | *Flow diagram visuals are provided in Figure 1A-B* |
|  |  | (c) Consider use of a flow diagram | *Flow diagram visuals are provided in Figure 1A-B* |
| Descriptive Data | 14 | (a) Give characteristics of study participants (e.g., demographic, clinical, social) and information on exposures and potential confounders | *Patient characteristics were included in Tables 1-5 as well as in the adjusted statistical models* |
|  |  | (b) Indicate number of participants with missing data for each variable of interest | *There is no data missing from participating patients as these patients were excluded prior to analysis* |
|  |  | (c) *Cohort study*—Summarize follow-up time (e.g., average and total amount) | *Not applicable* |
| Outcome Data | 15 | *Cohort study*—Report numbers of outcome events or summary measures over time | *Outcome events were reported in the Results* |
| Main Results | 16 | (*a*) Give unadjusted estimates and, if applicable, confounder-adjusted estimates and their precision (eg, 95% confidence interval). Make clear which confounders were adjusted for and why they were included | *Unadjusted and confounder-adjusted estimates were presented for all outcomes* |
|  |  | (*b*) Report category boundaries when continuous variables were categorized | *Not applicable* |
|  |  | (*c*) If relevant, consider translating estimates of relative risk into absolute risk for a meaningful time period | *Not applicable* |
| **Discussion** | | |  |
| Key Results | 18 | Summarize key results with reference to study objectives | *Key results summary with reference to study objectives is mentioned in paragraph 1 of the Discussion* |
| Limitations | 19 | Discuss limitations of the study, taking into account sources of potential bias or imprecision. Discuss both direction and magnitude of any potential bias | *The limitations of the study were outlined in paragraph 5 of the Discussion* |
| Interpretation | 20 | Give a cautious overall interpretation of results considering objectives, limitations, multiplicity of analyses, results from similar studies, and other relevant evidence | *Overall interpretation of results is explained in the last paragraph of the Discussion* |
| Generalizability | 21 | Discuss the generalizability (external validity) of the study results | *Expressed in paragraphs 2-4 of the Discussion* |
| **Other Information** | | |  |
| Funding | 22 | Give the source of funding and the role of the funders for the present study | *Grifols, SSNA* |
