# Supplementary material for: Antithrombin concentrates may benefit cardiopulmonary bypass patients with suspected heparin resistance: A retrospective analysis of real-world data
Source: Heliyon. 2023 Aug 29;9(9):e19497. doi: 10.1016/j.heliyon.2023.e19497 (PMC10558716; doi:10.1016/j.heliyon.2023.e19497)
Supplement: Multimedia component 2 [file mmc2.docx]

**Supplemental Tables**

**Supplemental Table 1: Procedure codes for identification of fresh frozen plasma patients**

| **Procedure Type** | **Procedure Code** | **Procedure Description** |
| --- | --- | --- |
| **Identification of fresh frozen plasma patients** | | |
| ICD9 | 99.07 | Transfusion of other serum |
| ICD10-PCS | 30230K0 | Transfusion of Autologous Frozen Plasma into Peripheral Vein, Open Approach |
| ICD10-PCS | 30230K1 | Transfusion of Nonautologous Frozen Plasma into Peripheral Vein, Open Approach |
| ICD10-PCS | 30233K0 | Transfusion of Autologous Frozen Plasma into Peripheral Vein, Percutaneous Approach |
| ICD10-PCS | 30233K1 | Transfusion of Nonautologous Frozen Plasma into Peripheral Vein, Percutaneous Approach |
| ICD10-PCS | 30240K0 | Transfusion of Autologous Frozen Plasma into Central Vein, Open Approach |
| ICD10-PCS | 30240K1 | Transfusion of Nonautologous Frozen Plasma into Central Vein, Open Approach |
| ICD10-PCS | 30243K0 | Transfusion of Autologous Frozen Plasma into Central Vein, Percutaneous Approach |
| ICD10-PCS | 30243K1 | Transfusion of Nonautologous Frozen Plasma into Central Vein, Percutaneous Approach |
| ICD10-PCS | 30250K0 | Transfusion of Autologous Frozen Plasma into Peripheral Artery, Open Approach |
| ICD10-PCS | 30250K1 | Transfusion of Nonautologous Frozen Plasma into Peripheral Artery, Open Approach |
| ICD10-PCS | 30253K0 | Transfusion of Autologous Frozen Plasma into Peripheral Artery, Percutaneous Approach |
| ICD10-PCS | 30253K1 | Transfusion of Nonautologous Frozen Plasma into Peripheral Artery, Percutaneous Approach |
| ICD10-PCS | 30260K0 | Transfusion of Autologous Frozen Plasma into Central Artery, Open Approach |
| ICD10-PCS | 30260K1 | Transfusion of Nonautologous Frozen Plasma into Central Artery, Open Approach |
| ICD10-PCS | 30263K0 | Transfusion of Autologous Frozen Plasma into Central Artery, Percutaneous Approach |
| ICD10-PCS | 30263K1 | Transfusion of Nonautologous Frozen Plasma into Central Artery, Percutaneous Approach |
| ICD10-PCS | 30273K1 | Transfusion of Nonautologous Frozen Plasma into Products of Conception, Circulatory, Percutaneous Approach |
| ICD10-PCS | 30277K1 | Transfusion of Nonautologous Frozen Plasma into Products of Conception, Circulatory, Via Natural or Artificial Opening |
| **Identification of cardiopulmonary bypass** | | |
| ICD9 | 39.61 | Extracorporeal circulation auxiliary to open-heart surgery |
| CPT4 | 33120 | Excision of intracardiac tumor, resection with cardiopulmonary bypass |
| CPT4 | 33305 | Repair of cardiac wound; with cardiopulmonary bypass |
| CPT4 | 33315 | Cardiotomy, exploratory (includes removal of foreign body, atrial or ventricular thrombus); with cardiopulmonary bypass |
| CPT4 | 33322 | Suture repair of aorta or great vessels; with cardiopulmonary bypass |
| CPT4 | 33335 | Insertion of graft, aorta or great vessels; with cardiopulmonary bypass |
| CPT4 | 33403 | Valvuloplasty, aortic valve; using transventricular dilation, with cardiopulmonary bypass |
| CPT4 | 33405 | Replacement, aortic valve, with cardiopulmonary bypass; with prosthetic valve other than homograft or stentless valve |
| CPT4 | 33406 | Replacement, aortic valve, with cardiopulmonary bypass; with allograft valve (freehand) |
| CPT4 | 33410 | Replacement, aortic valve, with cardiopulmonary bypass; with stentless tissue valve |
| CPT4 | 33422 | Valvotomy, mitral valve; open heart, with cardiopulmonary bypass |
| CPT4 | 33425 | Valvuloplasty, mitral valve, with cardiopulmonary bypass; |
| CPT4 | 33426 | Valvuloplasty, mitral valve, with cardiopulmonary bypass; with prosthetic ring |
| CPT4 | 33427 | Valvuloplasty, mitral valve, with cardiopulmonary bypass; radical reconstruction, with or without ring |
| CPT4 | 33430 | Replacement, mitral valve, with cardiopulmonary bypass |
| CPT4 | 33460 | Valvectomy, tricuspid valve, with cardiopulmonary bypass |
| CPT4 | 33465 | Replacement, tricuspid valve, with cardiopulmonary bypass |
| CPT4 | 33474 | Valvotomy, pulmonary valve, open heart; with cardiopulmonary bypass |
| CPT4 | 33496 | Repair of non-structural prosthetic valve dysfunction with cardiopulmonary bypass (separate procedure) |
| CPT4 | 33500 | Repair of coronary arteriovenous or arteriocardiac chamber fistula; with cardiopulmonary bypass |
| CPT4 | 33504 | Repair of anomalous coronary artery from pulmonary artery origin; by graft, with cardiopulmonary bypass |
| CPT4 | 33641 | Repair atrial septal defect, secundum, with cardiopulmonary bypass, with or without patch |
| CPT4 | 33702 | Repair sinus of Valsalva fistula, with cardiopulmonary bypass; |
| CPT4 | 33710 | Repair sinus of Valsalva fistula, with cardiopulmonary bypass; with repair of ventricular septal defect |
| CPT4 | 33720 | Repair sinus of Valsalva aneurysm, with cardiopulmonary bypass |
| CPT4 | 33736 | Atrial septectomy or septostomy; open heart with cardiopulmonary bypass |
| CPT4 | 33814 | Obliteration of aortopulmonary septal defect; with cardiopulmonary bypass |
| CPT4 | 33853 | Repair of hypoplastic or interrupted aortic arch using autogenous or prosthetic material; with cardiopulmonary bypass |
| CPT4 | 33860 | Ascending aorta graft, with cardiopulmonary bypass, with or without valve suspension; |
| CPT4 | 33864 | Ascending aorta graft, with cardiopulmonary bypass with valve suspension, with coronary reconstruction and valve-sparing aortic annulus remodeling (eg, David Procedure, Yacoub Procedure) |
| CPT4 | 33870 | Transverse arch graft, with cardiopulmonary bypass |
| CPT4 | 33910 | Pulmonary artery embolectomy; with cardiopulmonary bypass |
| CPT4 | 33916 | Pulmonary endarterectomy, with or without embolectomy, with cardiopulmonary bypass |
| CPT4 | 33922 | Transection of pulmonary artery with cardiopulmonary bypass |
| CPT4 | 33926 | Repair of pulmonary artery arborization anomalies by unifocalization; with cardiopulmonary bypass |

**Supplemental Table 2: Attrition tables for cohorts and methods**

| **Criteria** | **Unique Patients** | **Unique Visits** |
| --- | --- | --- |
| **Cohorts** | | |
| **FFP Cohort** | | |
| Visits selected from Methods F.1, F.2, and F.3 and CPB procedures (see below) | 279 | 281 |
| Visits that do not overlap with AT cohort | 275 | 277 |
| Inpatient encounter during index visit | 275 | 277 |
| Missing or unknown sex | 275 | 277 |
| Patient age 18 years old or older | 254 | 255 |
| Final FFP cohort | 254 | **255** |
| **AT Cohort** | | |
| Visits selected from Method A.1 and CPB procedures | 356 | 357 |
| Visits that do not overlap with FFP cohort | 353 | 353 |
| Missing or unknown sex | 353 | 353 |
| Patient age 18 years old or older | 247 | 247 |
| Final AT cohort | 247 | **247** |
| **Methods** | | |
| **Method F.1** | | |
| Patients with an FFP procedure code | 84,636 | 92,056 |
| Received heparin | 22,771 | 23,521 |
| ACT lab at least 2 min after receipt of heparin | 939 | 946 |
| ACT at least 2 min after heparin and ACT GE 150 and LE 400 | 493 | 496 |
| CPB procedure | 250 | **251** |
| **Method A.1** | | |
| Receipt of antithrombin III or antithrombin recombinant | 2,439 | 2,470 |
| Inpatient encounter during visit | 2,180 | 2,204 |
| Received heparin | 1,483 | 1,499 |
| CPB Procedure | 356 | **357** |
| **Method F.2** | | |
| FFP procedure | 84,636 | 92,056 |
| Received Heparin | 22,771 | 23,521 |
| Heparin per kg GE 300 | 3,126 | 3,202 |
| Has ACT at least 2 min after hep | 179 | 179 |
| ACT GE 150 AND ACT LE 400 | 110 | 110 |
| CPB Procedure | 56 | **56** |
| or | | |
| Received FFP | 84,636 | 92,056 |
| Received Heparin | 22,771 | 23,521 |
| Heparin per kg GE 400 | 2,311 | 2,357 |
| Has ACT at least 2 min after hep | 124 | 124 |
| ACT GE 150 AND ACT LE 480 | 97 | 97 |
| CPB procedure | 52 | **52** |
| **Method F.3** | | |
| FFP procedure | 84,636 | 92,056 |
| Received Heparin | 22,771 | 23,521 |
| Has a baseline ACT lab | 76 | 77 |
| Heparin Sensitivity Index LT 1.3 | 75 | 76 |
| CPB Procedure | 36 | **37** |

F.1, FFP Method 1; A.1, AT Method 1; F.2, FFP Method 2; F.3, FFP Method 3; ACT, activated clotting time; AT, antithrombin; CPB, cardiopulmonary bypass; FFP, fresh frozen plasma; GE, greater than or equal to; LE, less than or equal to; LT, less than.

**Supplemental Table 3: Additional patient and hospital characteristics**

| **Characteristic** | **AT (N=247)** | **FFP (N=255)** | ***P-*value*^a^*** |
| --- | --- | --- | --- |
| **Patient Characteristics** | | | |
| **Charlson Comorbidities** | | | |
| Myocardial infarction | 77 (31.2) | 95 (37.3) |  |
| Congestive heart failure | 111 (44.9) | 140 (54.9) |  |
| Peripheral vascular disease | 37 (15.0) | 70 (27.5) |  |
| Cerebrovascular disease | 36 (14.6) | 49 (19.2) |  |
| Dementia | 0 (0.0) | 1 (0.4) |  |
| Chronic pulmonary disease | 82 (33.2) | 104 (40.8) |  |
| Rheumatic disease | 5 (2.0) | 6 (2.4) |  |
| Peptic ulcer disease | 3 (1.2) | 4 (1.6) |  |
| Mild liver disease | 12 (4.9) | 26 (10.2) |  |
| Diabetes without chronic complication | 72 (29.1) | 84 (32.9) |  |
| Diabetes with chronic complication | 14 (5.7) | 21 (8.2) |  |
| Hemiplegia or paraplegia | 4 (1.6) | 4 (1.6) |  |
| Renal disease | 42 (17.0) | 79 (31.0) |  |
| Any malignancy, including lymphoma and leukemia, except malignant neoplasm of skin | 4 (1.6) | 13 (5.1) |  |
| Moderate or severe liver disease | 2 (0.8) | 3 (1.2) |  |
| Metastatic solid tumor | 1 (0.4) | 0 (0.0) |  |
| AIDS/HIV | 0 (0.0) | 3 (1.2) |  |
| **Hospital Characteristics** | | | |
| **Census Region** | | | |
| Northeast/South | 242 (98.0) | 175 (68.6) | *P* < .001 |
| Midwest/West | 5 (2.0) | 80 (31.4) |  |
| **Acute status of hospital** | | | |
| Acute | 247 (100.0) | 255 (100.0) |  |
| **Facility bed size** | | | |
| <500 | 28 (11.3) | 167 (65.5) | *P* < .001 |
| 500+ | 219 (88.7) | 88 (34.5) |  |
| **Teaching/non-teaching facility** | | | |
| Teaching | 240 (97.2) | 145 (56.9) | *P* < .001 |
| Non-teaching | 7 (2.8) | 110 (43.1) |  |
| **Urban/rural hospital** | | | |
| Urban | 239 (96.8) | 186 (72.9) | *P* < .001 |
| Rural | 8 (3.2) | 69 (27.1) |  |

Data are numbers (percentages) unless otherwise indicated. *^a^*T-tests were conducted for continuous data while chi-square tests were conducted for categorical data. AIDS, acquired immune deficiency syndrome; AT, antithrombin; FFP, fresh frozen plasma; HIV, human immunodeficiency virus.

**Supplemental Table 4: Mortality model (N=502)**

|  | **Model 1: Univariate Analyses** | | **Model 2: Patient Characteristics and Charlson*^a^*** | |
| --- | --- | --- | --- | --- |
| **Characteristic** | **OR (95% CI)** | ***P-*value** | **OR (95% CI)** | ***P-*value** |
| **AT or FFP** | | | | |
| AT | 0.29 (0.13-0.66) | *P* = .003 | 0.34 (0.15-0.80) | *P* = .01 |
| FFP | *Reference* |  | *Reference* |  |
| **Age, year** | 1.00 (0.97-1.03) | *P* = .93 | 1.00 (0.97-1.03) | *P* = .84 |
| **Age group** | | | | |
| 18-49 | 0.82 (0.27-2.50) | *P* = .73 | ― | ― |
| 50-64 | 0.99 (0.46-2.13) | *P* = .97 | ― | ― |
| 65+ | *Reference* |  | ― | ― |
| **Sex** | | | | |
| Male | *Reference* |  | *Reference* |  |
| Female | 3.49 (1.71-7.10) | *P* < .001 | 3.33 (1.59-6.97) | *P* = .002 |
| **Ethnicity** | | | | |
| Caucasian | *Reference* |  | *Reference* |  |
| African American | 2.38 (0.85-6.64) | *P* = .10 | 2.00 (0.68-5.92) | *P* = .21 |
| Other | 2.00 (0.78-5.14) | *P* = .15 | 1.49 (0.56-4.00) | *P* = .43 |
| **Admission source** | | | | |
| Physician or Clinical Referral | *Reference* |  | ― | ― |
| Hospital or Facility Transfer | 0.69 (0.28-1.71) | *P* = .43 | ― | ― |
| Emergency Room | 0.78 (0.32-1.93) | *P* = .59 | ― | ― |
| Not Specified | 0.50 (0.11-2.21) | *P* = .36 | ― | ― |
| **Admission type** | | | | |
| Emergency/Urgent | *Reference* |  | ― | ― |
| Elective | 1.10 (0.54-2.25) | *P* = .80 | ― | ― |
| Not Specified | 0.84 (0.11-6.62) | *P* = .87 | ― | ― |
| **Census Region** | | | | |
| Northeast/South | *Reference* |  | ― | ― |
| Midwest/West | 1.86 (0.83-4.13) | *P* = .13 | ― | ― |
| **Teaching/non-teaching facility** | | | | |
| Teaching | 0.31 (0.15-0.63) | *P* = .001 | ― | ― |
| Non-teaching | *Reference* |  | ― | ― |
| **Urban/rural hospital** | | | | |
| Urban | *Reference* |  | ― | ― |
| Rural | 2.12 (0.95-4.73) | *P* = .07 | ― | ― |
| **Charlson Comorbidities** | | | | |
| Charlson Comorbidity Index | 1.21 (1.06-1.39) | *P* = .004 | 1.16 (1.00-1.34) | *P* = .05 |
| Myocardial infarction | 1.05 (0.51-2.18) | *P* = .90 | ― | ― |
| Congestive heart failure | 1.29 (0.64-2.60) | *P* = .48 | ― | ― |
| Peripheral vascular disease | 2.46 (1.19-5.10) | *P* = .02 | ― | ― |
| Cerebrovascular disease | 2.55 (1.19-5.45) | *P* = .02 | ― | ― |
| Dementia | ― | ― | ― | ― |
| Chronic pulmonary disease | 1.56 (0.77-3.14) | *P* = .21 | ― | ― |
| Rheumatic disease | 5.56 (1.41-22.03) | *P* = .01 | ― | ― |
| Peptic ulcer disease | ― | ― | ― | ― |
| Mild liver disease | 2.27 (0.83-6.26) | *P* = .11 | ― | ― |
| Diabetes without chronic complication | 0.79 (0.36-1.73) | *P* = .55 | ― | ― |
| Diabetes with chronic complication | 2.52 (0.91-6.97) | *P* = .08 | ― | ― |
| Hemiplegia or paraplegia | 2.00 (0.24-16.71) | *P* = .52 | ― | ― |
| Renal disease | 2.06 (1.00-4.26) | *P* = .05 | ― | ― |
| Any malignancy, including lymphoma and leukemia, except malignant neoplasm of skin | 1.89 (0.41-8.62) | *P* = .41 | ― | ― |
| Moderate or severe liver disease | 3.52 (0.38-32.35) | *P* = .27 | ― | ― |
| Metastatic solid tumor | ― | ― | ― | ― |
| AIDS/HIV | ― | ― | ― | ― |
| **APS score** | 1.07 (1.04-1.10) | *P* < .001 | ― | ― |

*^a^*Logistic regression with mortality as a binary outcome was used in Model 2. AIDS, acquired immune deficiency syndrome; APS, acute physiology score; AT, antithrombin; CI, confidence interval; FFP, fresh frozen plasma; HIV, human immunodeficiency virus; OR, odds ratio.

**Supplemental Table 5: Hospital free days model (N=502)**

|  | **Model 1: Univariate Analysis** | | **Model 2: Patient Characteristics and Charlson*^a^*** | |
| --- | --- | --- | --- | --- |
| **Characteristic** | **Hospital Free Days Ratio (95% CI)** | ***P-*value** | **Hospital Free Days Ratio (95% CI)** | ***P-*value** |
| **AT or FFP** | | | | |
| AT | 1.10 (1.03-1.18) | *P* = .004 | 1.06 (0.99-1.13) | *P* = .08 |
| FFP | *Reference* |  | *Reference* |  |
| **Age, year** | 1.00 (1.00-1.00) | *P* = .86 | 1.00 (1.00-1.00) | *P* = .56 |
| **Age group** | | | | |
| 18-49 | 0.97 (0.87-1.07) | *P* = .50 | ― | ― |
| 50-64 | 1.02 (0.94-1.10) | *P* = .66 | ― | ― |
| 65+ | *Reference* |  | ― | ― |
| **Sex** | | | | |
| Male | *Reference* |  | *Reference* |  |
| Female | 0.97 (0.90-1.05) | *P* = .43 | 0.97 (0.91-1.05) | *P* = .49 |
| **Ethnicity** | | | | |
| Caucasian | *Reference* |  | *Reference* |  |
| African American | 0.86 (0.76-0.98) | *P* = .03 | 0.89 (0.78-1.01) | *P* = .06 |
| Other | 0.99 (0.89-1.10) | *P* = .81 | 1.02 (0.92-1.13) | *P* = .72 |
| **Admission source** | | | | |
| Physician or Clinical Referral | *Reference* |  | ― | ― |
| Hospital or Facility Transfer | 0.99 (0.91-1.08) | *P* = .79 | ― | ― |
| Emergency Room | 0.95 (0.87-1.04) | *P* = .30 | ― | ― |
| Not Specified | 0.94 (0.84-1.06) | *P* = .34 | ― | ― |
| **Admission type** | | | | |
| Emergency/Urgent | *Reference* |  | ― | ― |
| Elective | 1.04 (0.97-1.11) | *P* = .33 | ― | ― |
| Not Specified | 1.07 (0.90-1.29) | *P* = .44 | ― | ― |
| **Census Region** | | | | |
| Northeast/South | *Reference* |  | ― | ― |
| Midwest/West | 0.91 (0.83-0.99) | *P* = .03 | ― | ― |
| **Teaching/non-teaching facility** | | | | |
| Teaching | 1.05 (0.97-1.14) | *P* = .21 | ― | ― |
| Non-teaching | *Reference* |  | ― | ― |
| **Urban/rural hospital** | | | | |
| Urban | *Reference* |  | ― | ― |
| Rural | 0.89 (0.81-0.98) | *P* = .02 | ― | ― |
| **Charlson Comorbidities** | | | | |
| Charlson Comorbidity Index | 0.95 (0.94-0.97) | *P* < .001 | 0.95 (0.94-0.97) | *P* < .001 |
| Myocardial infarction | 0.96 (0.90-1.03) | *P* = .28 | ― | ― |
| Congestive heart failure | 0.94 (0.88-1.01) | *P* = .07 | ― | ― |
| Peripheral vascular disease | 0.91 (0.84-0.99) | *P* = .04 | ― | ― |
| Cerebrovascular disease | 0.85 (0.78-0.94) | *P* < .001 | ― | ― |
| Dementia | 0.84 (0.41-1.70) | *P* = .62 | ― | ― |
| Chronic pulmonary disease | 0.90 (0.84-0.96) | *P* = .002 | ― | ― |
| Rheumatic disease | 0.99 (0.75-1.29) | *P* = .91 | ― | ― |
| Peptic ulcer disease | 0.89 (0.68-1.17) | *P* = .41 | ― | ― |
| Mild liver disease | 0.84 (0.72-0.98) | *P* = .02 | ― | ― |
| Diabetes without chronic complication | 0.97 (0.90-1.04) | *P* = .40 | ― | ― |
| Diabetes with chronic complication | 0.89 (0.77-1.03) | *P* = .11 | ― | ― |
| Hemiplegia or paraplegia | 0.65 (0.49-0.87) | *P* = .004 | ― | ― |
| Renal disease | 0.85 (0.78-0.92) | *P* < .001 | ― | ― |
| Any malignancy, including lymphoma and leukemia, except malignant neoplasm of skin | 0.75 (0.61-0.92) | *P* = .006 | ― | ― |
| Moderate or severe liver disease | 0.61 (0.43-0.87) | *P* = .007 | ― | ― |
| Metastatic solid tumor | ― | ― | ― | ― |
| AIDS/HIV | 0.69 (0.46-1.03) | *P* = .07 | ― | ― |
| **APS score** | 0.99 (0.99-1.00) | *P* = .004 | ― | ― |

*^a^*GLMs with gamma distribution and log link function was used in Model 2. AIDS, acquired immune deficiency syndrome; APS, acute physiology score; AT, antithrombin; CI, confidence interval; FFP, fresh frozen plasma; GLM, generalized linear model; HIV, human immunodeficiency virus.

**Supplemental Table 6: Intensive care unit length of stay model (N=278)**

|  | **Model 1: Univariate Analyses** | | **Model 2: Patient Characteristics and Charlson*^a^*** | |
| --- | --- | --- | --- | --- |
| **Characteristic** | **ICU LOS Ratio (95% CI)** | ***P-*value** | **ICU LOS Ratio (95% CI)** | ***P-*value** |
| **AT or FFP** | | | | |
| AT | 0.78 (0.63-0.96) | *P* = .02 | 0.93 (0.73-1.17) | *P* = .53 |
| FFP | *Reference* |  | *Reference* |  |
| **Age, year** | 1.01 (1.00-1.01) | *P* = .16 | 1.00 (1.00-1.01) | *P* = .35 |
| **Age group** | | | | |
| 18-49 | 1.10 (0.80-1.50) | *P* = .56 | ― | ― |
| 50-64 | 0.86 (0.68-1.10) | *P* = .23 | ― | ― |
| 65+ | *Reference* |  | ― | ― |
| **Sex** | | | | |
| Male | *Reference* |  | *Reference* |  |
| Female | 1.30 (1.04-1.63) | *P* = .02 | 1.21 (0.97-1.51) | *P* = .09 |
| **Ethnicity** | | | | |
| Caucasian | *Reference* |  | *Reference* |  |
| African American | 0.86 (0.55-1.34) | *P* = .51 | 0.92 (0.59-1.41) | *P* = .69 |
| Other | 1.07 (0.77-1.49) | *P* = .67 | 1.15 (0.84-1.58) | *P* = .39 |
| **Admission source** | | | | |
| Physician or Clinical Referral | *Reference* |  | ― | ― |
| Hospital or Facility Transfer | 1.01 (0.78-1.31) | *P* = .94 | ― | ― |
| Emergency Room | 1.35 (1.04-1.76) | *P* = .03 | ― | ― |
| Not Specified | 1.17 (0.76-1.79) | *P* = .48 | ― | ― |
| **Admission type** | | | | |
| Emergency/Urgent | *Reference* |  | ― | ― |
| Elective | 0.70 (0.56-0.88) | *P* = .002 | ― | ― |
| Not Specified | 0.99 (0.63-1.56) | *P* = .97 | ― | ― |
| **Census Region** | | | | |
| Northeast/South | *Reference* |  | ― | ― |
| Midwest/West | 1.47 (1.13-1.92) | *P* = .005 | ― | ― |
| **Teaching/non-teaching facility** | | | | |
| Teaching | 0.76 (0.59-0.97) | *P* = .03 | ― | ― |
| Non-teaching | *Reference* |  | ― | ― |
| **Urban/rural hospital** | | | | |
| Urban | *Reference* |  | ― | ― |
| Rural | 1.65 (1.30-2.11) | *P* < .001 | ― | ― |
| **Charlson Comorbidities** | | | | |
| Charlson Comorbidity Index | 1.13 (1.08-1.18) | *P* < .001 | 1.12 (1.06-1.17) | *P* < .001 |
| Myocardial infarction | 1.13 (0.91-1.42) | *P* = .27 | ― | ― |
| Congestive heart failure | 1.37 (1.11-1.69) | *P* = .003 | ― | ― |
| Peripheral vascular disease | 1.32 (1.01-1.74) | *P* = .047 | ― | ― |
| Cerebrovascular disease | 1.22 (0.90-1.64) | *P* = .20 | ― | ― |
| Dementia | 0.59 (0.10-3.41) | *P* = .55 | ― | ― |
| Chronic pulmonary disease | 1.37 (1.10-1.71) | *P* = .005 | ― | ― |
| Rheumatic disease | 1.08 (0.52-2.24) | *P* = .83 | ― | ― |
| Peptic ulcer disease | 1.00 (0.41-2.43) | *P* = .99 | ― | ― |
| Mild liver disease | 1.97 (1.31-2.97) | *P* = .001 | ― | ― |
| Diabetes without chronic complication | 1.13 (0.90-1.43) | *P* = .28 | ― | ― |
| Diabetes with chronic complication | 1.24 (0.82-1.86) | *P* = .31 | ― | ― |
| Hemiplegia or paraplegia | 3.60 (1.66-7.82) | *P* = .001 | ― | ― |
| Renal disease | 1.32 (1.03-1.69) | *P* = .03 | ― | ― |
| Any malignancy, including lymphoma and leukemia, except malignant neoplasm of skin | 2.20 (1.32-3.67) | *P* = .003 | ― | ― |
| Moderate or severe liver disease | 1.74 (0.30-10.11) | *P* = .54 | ― | ― |
| Metastatic solid tumor | ― | ― | ― | ― |
| AIDS/HIV | 1.23 (0.35-4.30) | *P* = .74 | ― | ― |
| **APS score** | 1.01 (1.00-1.02) | *P* = .10 | ― | ― |

*^a^*GLMs with gamma distribution and log link function was used in Model 2. AIDS, acquired immune deficiency syndrome; APS, acute physiology score; AT, antithrombin; CI, confidence interval; FFP, fresh frozen plasma; GLM, generalized linear model; HIV, human immunodeficiency virus; ICU, intensive care unit; LOS, length of stay.
